# New aspects in snake venom toxicology

**DOI:** 10.1007/s00204-021-03066-4

**Published:** 2021-05-06

**Authors:** Hermann M. Bolt

**Affiliations:** Leibniz Research Centre for Working Environment and Human Factors (IfADo), Ardeystr. 67, 44139 Dortmund, Germany

Snakebite envenomation causes > 81,000 deaths and incapacities in another 400,000 people worldwide every year. Snake venoms are complex natural secretions comprised of hundreds of different molecules with a wide range of biological functions, which after injection cause local and systemic toxic manifestations (Di Nicola 2021; Acunha et al 2021).

Against this background, snake venom toxicology is a field of high toxicological impact that has attracted increased interest over the last decade. This is reflected by publications in Archives of Toxicology. Traditionally, toxicologists were primarily interested in snake venom effects on blood coagulation (Klöcking and Hoffmann 1991; Wiwanitkit 2006; Sanchez et al. 2009), a relevant field that continues as an important research matter (Teixeira et al 2011, Giron et al. 2013; Zheng et al. 2013, Sartim et al 2016). Other aspects are cytotoxicity (El Hakim 2011), neurotoxixity (Floriano et al 2019), and immunological effects (Silva da Franca et al. 2021) of snake venom constituents.

The scientific focus of recent articles has broadened, as clinical (Abd El-Aziz et al 2020) and pharmacological matters (Lee et al 2016) were incorporated. The latter aspect is highlighted in current reviews as a cutting-edge topic (Bordon et al 2020; Kalita et al 2021; Akhtar et al 2021). Here, the potential of snake venom constituents for cancer treatment is particularly promising. In this context, progress into identification of constituents of snake venom proteomes is important (Estrella et al 2011; Igci and Demiralp 2012).

The majority of studies have focused on the protein portion (toxins), without paying significant attention to other fractions. A heretofore neglected field is that of lipid constituents of snake venoms. A breakthrough in this new area was just reported in this journal. Acunha et al (2021) reported about an untargeted lipidomic approach, based on liquid chromatography with high-resolution mass spectrometry. This was applied to investigate the lipid constituents of venoms of the South American snakes *Crotalus durissus terrificus* and *Bothrops moojeni*. Phosphatidylcholines (PC), Lyso-PCs, phosphatidylethanolamines (PE), Lyso-PE, phosphatidylserine, phosphatidylinositol, ceramides, and sphingomyelin species were detected. The lipids included bioactive compounds such as platelet-activating factor (PAF) precursor, PAF-like molecules, plasmalogens, ceramides, and sphingomyelins with long fatty acid chain lengths, which may be associated with the systemic responses triggered by *Crotalus durissus terrificus* and *Bothrops moojeni* envenomation. The responses include platelet aggregation, activation of intercellular adhesion molecule 1 (ICAM1), apoptosis, as well as the production of pro-inflammatory lipid mediators, cytokines, and reactive species. The new lipidomics aspects will contribute to an increased understanding of the complex pathology elicited by snakebite envenomation.
